# Effect of Nitrate, Ammonium and Urea on Growth and Pinnatoxin G Production of *Vulcanodinium rugosum*

**DOI:** 10.3390/md13095642

**Published:** 2015-09-02

**Authors:** Eric Abadie, Lamia Kaci, Tom Berteaux, Philipp Hess, Véronique Sechet, Estelle Masseret, Jean Luc Rolland, Mohamed Laabir

**Affiliations:** 1 Laboratoire Environnement Ressources du Languedoc Roussillon, Center for Marine Biodiversity, Exploitation and Conservation (MARBEC), Ifremer, Sète Cedex 3 CS30171, France; E-Mails: kaci_lamia@hotmail.fr (L.K.); tom.berteaux@ifremer.fr (T.B.); 2 Département ODE/UL/PHYC, Ifremer, Rue de l’Ile d’Yeu, Nantes Cedex 3 BP 21105 44311, France; E-Mails: philipp.hess@ifremer.fr (P.H.); veronique.sechet@ifremer.fr (V.S.); 3 Center for Marine Biodiversity, Exploitation and Conservation (MARBEC), Université de Montpellier, CNRS, IRD, Ifremer, Place Eugène Bataillon, CC93, Montpellier Cedex 5 34095, France; E-Mails: estelle.masseret@univ-montp2.fr (E.M.); mohamed.laabir@univ-montp2.fr (M.L.); 4 UMR 5244, Interaction Hotes Pathogenes Environnement (IHPE), Ifremer, Université de Perpignan, Université de Montpellier, Place Eugène Bataillon, CC80, Montpellier Cedex F-34095, France; E-Mail: jean.luc.rolland@ifremer.fr

**Keywords:** *Vulcanodinium rugosum*, pinnatoxin G, Ingril lagoon, growth, nitrogen source

## Abstract

*Vulcanodinium rugosum*, a recently described dinoflagellate species producing a potent neurotoxin (pinnatoxin G), has been identified in French Mediterranean lagoons and was responsible for recurrent episodes of shellfish toxicity detected by mouse bioassay. Until now, the biology and physiology of *V. rugosum* have not been fully investigated. We studied the growth characteristics and toxicity of a *V. rugosum* strain (IFR-VRU-01), isolated in the Ingril lagoon in June 2009 (North-Western French Mediterranean Sea). It was cultivated in Enriched Natural Sea Water (ENSW) with organic (urea) and inorganic (ammonium and nitrate) nitrogen, at a temperature of 25 °C and irradiance of 100 μmol/m^2^·s^−1^. Results showed that ammonium was assimilated by cells more rapidly than nitrate and urea. *V. rugosum* is thus an osmotrophic species using urea. Consequently, this nitrogen form could contribute to the growth of this dinoflagellate species in the natural environment. There was no significant difference (Anova, *p* = 0.856) between the growth rate of *V. rugosum* cultivated with ammonium (0.28 ± 0.11 day^−1^), urea (0.26 ± 0.08 day^−1^) and nitrate (0.24 ± 0.01 day^−1^). However, the production of chlorophyll *a* and pinnatoxin G was significantly lower with urea as a nitrogen source (Anova, *p* < 0.027), suggesting that nutritional conditions prevailing at the moment of the bloom could determine the cellular toxicity of *V. rugosum* and therefore the toxicity measured in contaminated mollusks. The relatively low growth rate (≤0.28 day^−1^) and the capacity of this species to continuously produce temporary cysts could explain why cell densities of this species in the water column are typically low (≤20,000 cells/L).

## 1. Introduction

Many photosynthetic microorganisms are able to develop massive blooms when environmental conditions are favorable. Some microalgal species are harmful and could produce toxins negatively affecting the functioning of the ecosystem and human health. These blooms are commonly called Harmful Algal Blooms(HABs) [1]. Various gastric or neurological syndromes are observed in humans following the consumption of contaminated shellfish [2,3]. Toxins can accumulate in the tissues of many species of bivalve mollusks (oysters, mussels), birds, and marine mammals [4]. HABs can have negative impacts on shellfish aquaculture, fish farming, and tourism. The exponential increase in the number of HABs in the world may be a result of growing exploitation of coastal waters for aquaculture, inducing an enrichment in organic matter, transfer through ballast water or via translocation of mollusks [5,6], and nutrient enrichment [7].

**Figure 1:**
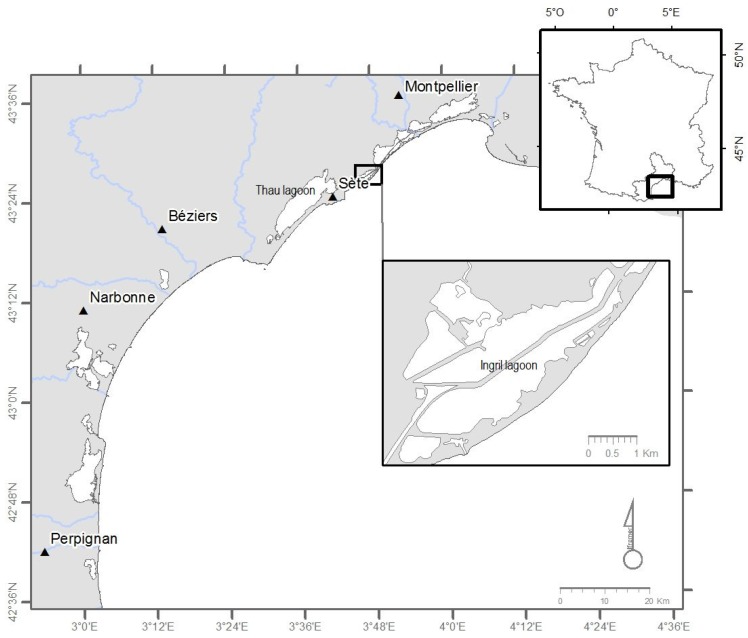
Ingril, a French Mediterranean lagoon.

Pinnatoxins were first characterized in the bivalve *Pinna muricata* Linnaeus in Japan [8]. Pinnatoxins E and F were detected in Pacific oysters in 2007 in Rangaunu harbor, Northland and New Zealand, whereas pinnatoxins E, F and G were found in the digestive gland of *Crassostrea gigas* from South Australia [9]. However, the causative organism producing these toxins was unknown. Rhodes *et al*. [10] isolated and cultured non-motile cells of an unidentified dinoflagellate present in New Zealand, responsible for the production of pinnatoxins. Soon after, from water samples collected from the Ingril lagoon, a morphologically very similar taxon was described by Nezan and Chomérat [11] as *Vulcanodinium rugosum*. A phylogenetic study based on Large Sub Unit ribosomal DNA (LSU rDNA) sequences confirmed that this taxon was new and belonged to the order of Peridiniales. A new species was proposed, *V. rugosum,* currently placed in the order of Peridiniales. This species was responsible for an unusual toxicity observed in the Ingril Lagoon (Figure 1) with particularly high concentrations of toxins for several months [12]. This study also clarified that *V. rugosum* produces pinnatoxin G (PnTX-G) in culture. Toxicity tests performed on mice with extracts of shellfish fed with *V. rugosum* cells revealed the presence of this pinnatoxin causing neurological symptoms [9,10,11,12,13]. The presence of this species was subsequently confirmed in Australia, New Zealand, Japan, Hawaii, China and the Tropical Mexican Pacific [13,14,15,16,17]. Strains isolated from different areas do not necessarily produce the same analogues. Strains from Australia and New Zealand produce pinnatoxins E and F [10,11,12,13]. Some Australian strains additionally produce pinnatoxin G, while the Japanese strains so far appear to exclusively produce pinnatoxin G [18], as is the case with the French strain for the type species [12].

Nutrients are among the most important factors controlling phytoplankton growth. Nitrogen (N) and phosphorus (P) play a crucial role in the growth and development of these micro-organisms. Eutrophication could be responsible for some HAB events [3,4,5,6,7,8,9,10,11,12,13,14,15,16,17,18,19]. Nutrient concentrations and ratios may influence cell growth as well as the toxicity of the micro-organisms [20,21,22,23]. Many dinoflagellates species use mixotrophy, which may give them a competitive advantage in marine ecosystems receiving organic input [7]. The use of dissolved organic matter (osmotrophy) and/or predation (phagotrophy) could be an important mode of nutrition for protists in oligotrophic ecosystems [24,25] or eutrophic estuaries [26,27]. For instance, a bloom of *Cochlodinium* sp. acquired an estimated ~55%–62% of its N supply from urea [28]. The importance of urea relative to the total dissolved organic nitrogen was 3%–25% in the Thau lagoon, France [29] and in the same range in a shallow eutrophic bay in Japan [30]. Collos *et al.* [29] showed that nitrate and nitrite contributed 0.1%–14% and 0.1%–5%, respectively, of growth requirements of *Alexandrium catenella* in the Thau lagoon, whereas ammonium and urea were the main N sources fueling growth of this harmful dinoflagellate (30%–100% and 2%–59%, respectively). Until now, no data on the effect of the nutrients on the physiology of *V. rugosum* had been available. This study aims to investigate the effect of different nitrogen sources, including inorganic nitrogen (nitrate, ammonium) and organic nitrogen (urea), on the morphology, growth, and toxicity of *V. rugosum* developing in the Ingril lagoon, a Mediterranean lagoon close to Thau, in the northwestern French Mediterranean Sea.

## 2. Results and Discussion

### 2.1. Cell Growth

**Figure 2:**
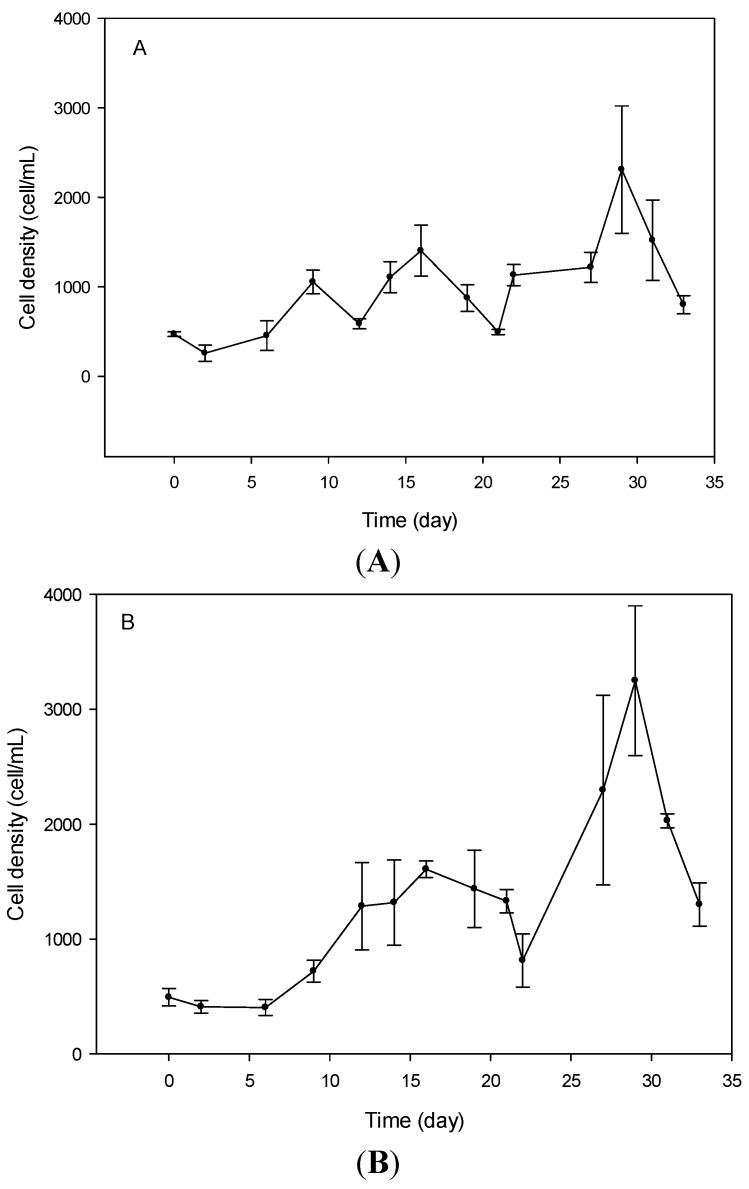

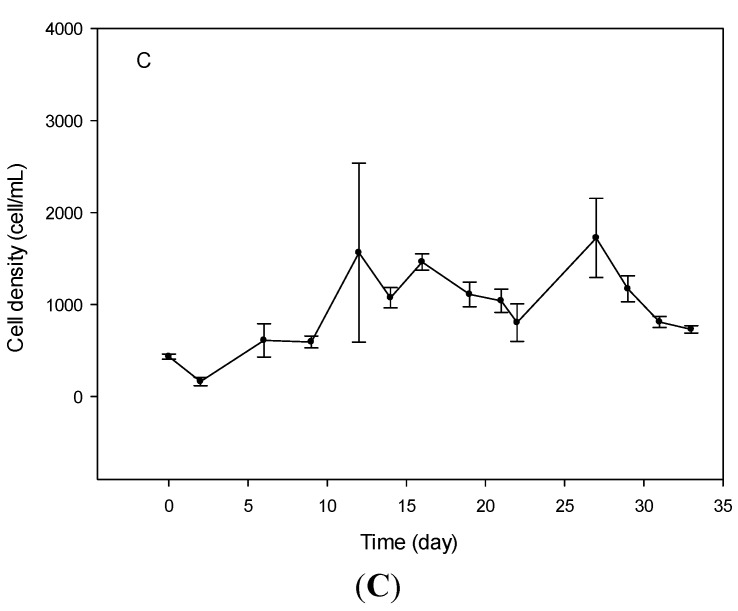
Growth curves of *Vulcanodinium rugosum* grown with nitrate (**A**); ammonium (**B**) and urea (**C**) as nitrogen sources.

For *V. rugosum* cultures grown with nitrate as the nitrogen source, the initiation phase was circa 2 days, whereas it was 6 days for cultures grown with urea or ammonium. There was no significant difference (Anova, *p* = 0.856) between the growth rate of *V. rugosum* cultivated with ammonium (0.28 ± 0.11 day^−1^), urea (0.26 ± 0.08 day^−1^), or nitrate (0.24 ± 0.01 day^−1^) as nitrogen source. For nitrate and ammonium, a rapid growth phase started on day 21 or 22 of the experiment, lasted for 5 days, and ended at the day 27 or 28, immediately followed by a significant drop in cell density (Figure 2A,B). For cultures grown on urea, we observed a significant increase in cell density from day 2 to day 12, reaching 1500 cells mL^−1^, followed by a progressive decrease in cell density. Then, we observed an increase of cell density from day 22 to day 27, with a maximum cell concentration of 1750 cells/mL (Figure 2C). This evolution was followed by a sharp decrease in cell concentration, as was observed for the other cultures grown with nitrate and ammonium.

In many ecosystems, and particularly in those with strong anthropogenic inputs, nutrients may vary quantitatively and qualitatively, depending on the degree of eutrophication or oligotrophication, thus influencing the development of dinoflagellates [7,29,31]. Until now, data on the growth characteristics, nutritional preferences, and toxicity of *V. rugosum* had not been available. Yet, such studies are required to understand the relationship between environmental parameters, e.g., nutritional factors prevailing in the environment, on one hand, and the growth and toxicity of this harmful dinoflagellate on the other. Here, we investigated the influence of inorganic (nitrate and ammonium) and organic (urea) dissolved nitrogen on the biology and toxin content of *V. rugosum*. This study showed that this dinoflagellate developing in the Ingril lagoon exhibited a low growth rate with all nitrogen sources and a temperature and irradiance of 25 °C and 100 μmol/m^2^·s^−1^ (0.24–0.28 day^−1^) compared to *Alexandrium catenella* growing in similar environmental conditions in the Thau lagoon (0.4–1 day^−1^) [32,33]. Nevertheless, the average growth rate of *V. rugosum* was closer to those found for benthic species such as *Ostreopsis* sp. (0.17–0.49 day^−1^) [34,35,36] and *Prorocentrum lima* (0.34 day^−1^) [37]. Maximum cell concentration (cell yield) reached in the laboratory did not exceed 3000 cells/mL for *V. rugosum*. This behavior appeared to be coherent with the low cell abundances (maximum of 20,000 cells/L) of *V. rugosum* measured in the water column in the Ingril lagoon during a monthly survey performed from April 2012 to May 2013 [38].

### 2.2. Cell Morphometry

**Figure 3:**
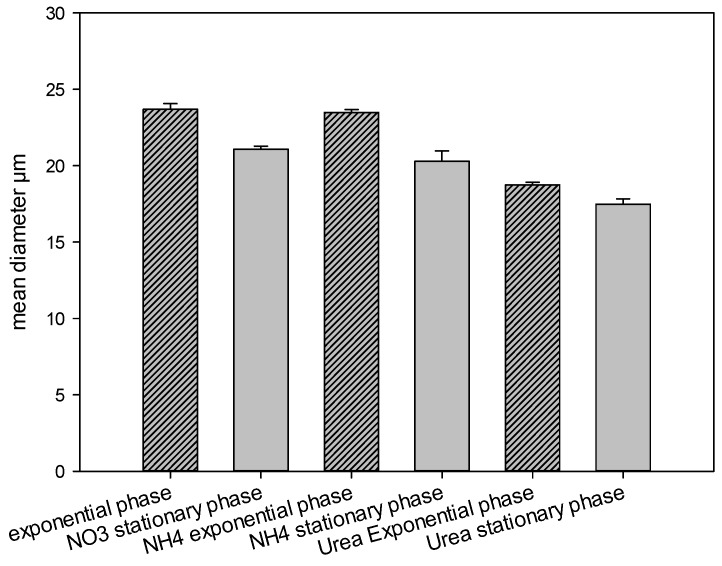
Mean diameter of *Vulcanodinium rugosum* cells grown on nitrate, ammonium, and urea, measured at exponential and stationary phases of growth, *n* ≥ 30.

For all nitrogen sources tested, the average cell diameter was larger at the exponential growth phase (23.7 ± 0.37 μm, 23.5 ± 0.18 μm, and 18.7 ± 0.18 μm for nitrate, ammonium, and urea, respectively) than that at the stationary phase (21 ± 0.21 μm, 20.3 ± 0.67 μm, and 17.5 ± 0.37 μm for nitrate, ammonium, and urea, respectively). There was no significant difference in average size between cells from cultures using ammonium compared to those grown using nitrate (one way ANOVA, *p* = 1). However, the average diameter of cells grown with urea was significantly lower (Anova, *p* < 0.001) than that of cells growing on nitrate and ammonium (Figure 3).

### 2.3. Variation of Nitrogen Concentration in Cultures

**Figure 4:**
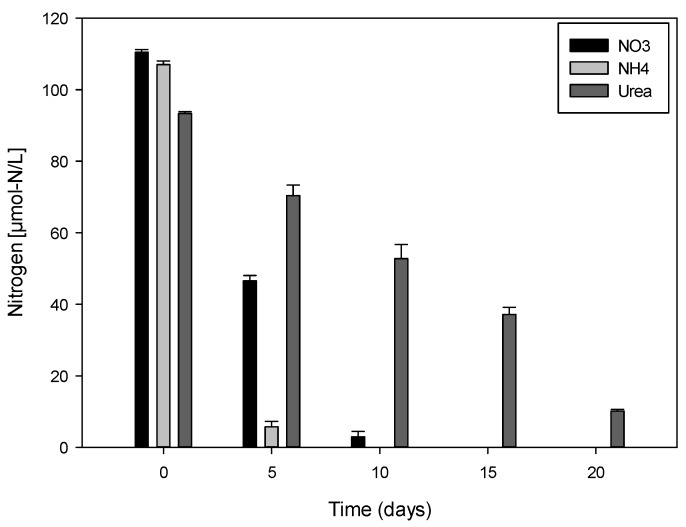
Variations of nitrate, ammonium, and urea concentrations (μmol-N/L) in the cultures of *Vulcanodinium rugosum*.

Ammonium concentration in cultures decreased rapidly until exhaustion after only one week (Figure 4). The initial concentration corresponds to the concentration of nitrogen in the inoculated media at the beginning of the experiment. Complete depletion in nitrate in culture media was observed after 12 days of culture. Ammonium was used up even more rapidly with concentrations not exceeding 5 μmol-N/L at day 6. *V. rugosum* was able to absorb urea as a nitrogen source and is thus an osmotrophic dinoflagellate. Urea concentration in cultures decreased progressively to circa 10 μmol-N/L after 20 days. Nitrogen uptake was estimated, taking into consideration the decrease in nitrogen concentration and increase in cell densities in cultures over time. Ammonium, nitrate, and urea were absorbed at a mean rate of 38.75, 18.74, and 5.46 μmol-N × 10^−6^/cell day^−1^, respectively.

We investigated the effect of different inorganic forms (nitrate and ammonium) and organic (urea) nitrogen, some of the major nutritive resources that could influence the growth and the cell yield of *V. rugosum*. All sources of nitrogen can be used by this species, suggesting the osmotrophic behavior of *V. rugosum*. This behavior may give dinoflagellates a competitive advantage *in situ* since organic nutrients such as urea could represent a significant source of nitrogen [7,29,39,40]. It has been shown that ammonium is rapidly assimilated by dinoflagellates because of the low energy cost required for protein synthesis [41]. Our results confirm these observations for the Mediterranean strain of *V. rugosum*. Compared to nitrate and urea, ammonium was rapidly assimilated by this dinoflagellate. Jauzein [42] showed that the absorption of urea by *A. catenella* resulted in intense excretion of ammonium into the culture medium. Cells primarily using ammonium may partly explain the slow uptake of urea (5.46 μmol-N × 10^−6^/cell day^−1^) compared to other nitrogen sources. Several studies have shown that the effects of organic and inorganic nitrogen differ greatly from one species to another and sometimes even from one strain to another for the same species [43]. Nutrients assimilated by phytoplankton species can influence both cell growth and toxin production [20,21,22,23,44]. Significant differences were shown to depend on the species, e.g., in the genus Alexandrium. For example, *A. catenella* primarily uses ammonium for growth [39]. *V. rugosum* cell density increased after the 22nd day of incubation despite nitrogen deficiency in all of the cultures. We therefore suggest that after senescence, dead cells may represent a source of particulate organic matter, which could be converted into dissolved inorganic nutrients by heterotrophic bacteria as our culture was not axenic. Inorganic nitrogen bacterially produced can be used by living cells, which would again promote growth [45].

### 2.4. Toxin Concentration Variations

**Figure 5:**
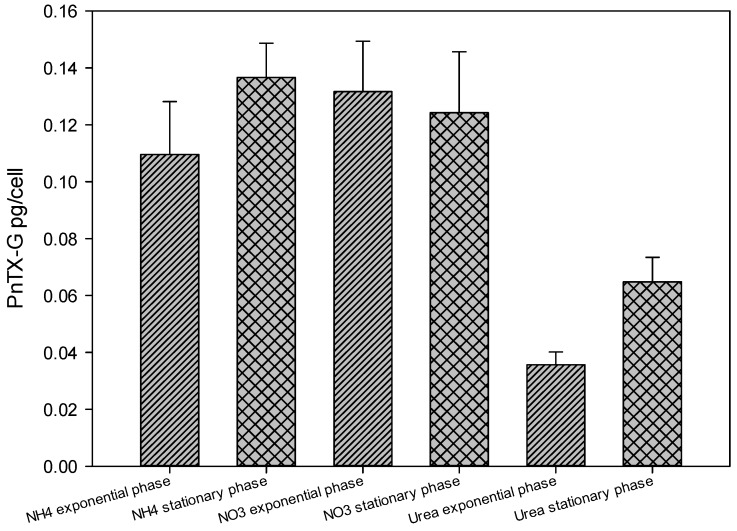
Concentration of pinnatoxin G in *Vulcanodinium rugosum* grown with nitrate, ammonium, and urea as nitrogen sources, at exponential and stationary phases of the growth cycle.

Concentrations of PnTX-G in *V.rugosum* cells, expressed in pg/cell, were measured both at the exponential and the stationary phases in cultures using different nitrogen sources. The use of urea had an impact on the production of PnTX-G. The toxin concentration in cells grown on this organic nitrogen form was significantly lower than that of cells grown on nitrate and ammonium (Figure 5, one-way ANOVA, *p* < 0.001). We also observed that PnTX-G production may depend on the growth phase. There was no significant difference in the amount of PnTX-G in cells grown on nitrate compared to those cultivated on ammonium (one way ANOVA, *p* > 0.35).

For comparison, a toxic strain of *A. tamarense* showed a high growth rate using urea as the nitrogen source, whereas the saxitoxin production rate was favored by nitrate [20,21,22,23]. In our study, the growth rate was not significantly different for *V. rugosum* when cultured with either urea or ammonium. However, the PnTX-G cell count was higher in cultures grown with inorganic nitrogen (ammonium and nitrate) than in cultures grown with urea.

### 2.5. Chlorophyll a Measurements

**Figure 6:**
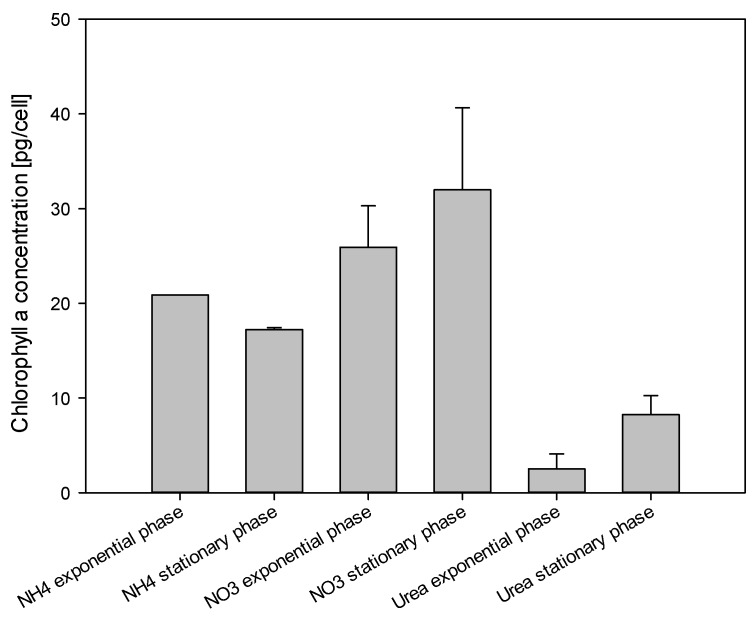
Chlorophyll *a* concentration in *Vulcanodinium rugosum* cells grown with nitrate, ammonium, and urea and harvested in the exponential and stationary phases of growth.

The chlorophyll a (Chl *a*) concentration (pg/cell) was measured in cultures with different nitrogen sources at exponential and stationary growth phases. Chl *a* concentration was significantly lower for *V. rugosum* cells grown on urea than for cells using ammonium or nitrate (one way ANOVA, *p* < 0.004). The highest concentration was detected in the cultures with nitrate and in cells harvested during the stationary phase with 32 ± 9 pg/cell. The lowest concentration was found in cultures using urea in the exponential phase of growth with 2.5 ± 1.6 pg/cell (Figure 6).

## 3. Experimental Section

### 3.1. Cultures of Vulcanodinium rugosum

The strain of *V. rugosum* (IFR-VRU-01) used in this study was isolated from a single vegetative cell sampled from the water column of the Ingril lagoon (Figure 1) in 2009. Water sampling was performed in 2009 during an important development for this species *in situ* (~2200 cells/L); the water temperature was 23.3 °C and salinity 36.5. The Ingril lagoon has a surface area of 685 hectares and communicates with the sea through Frontignan harbor, through a channel named Grau. Its depth reaches a maximum of 1.20 m with a sandy bottom (Service Maritime de Navigation du Languedoc Roussillon (SMNLR), Saint-Gilles, France, 2006). *V. rugosum* was grown on an Enriched Natural Sea Water (ENSW) culture medium [46]. It was based on water sampled *in situ*, filtered at 0.2 μm, and then autoclaved. Nutrients and trace elements were added to all the water at time zero t_0_ (batch culture). This strain was maintained in culture through monthly subcultures. In previous laboratory experiments, we determined the optimal growth conditions for this dinoflagellate, which corresponded to a temperature of 25 ± 1 °C, salinity of 36, light intensity of 100 μmol/m^2^·s^−1^ using cool white fluorescent light, and a photoperiod of 12 h/12 h. Small-scale turbulence negatively affects the growth of dinoflagellates inducing the formation of temporary cysts, therefore agitation prior to sampling was very moderate.

As described by Rhodes *et al.* [18], the *V. rugosum* life cycle shows typical motile vegetative cells and unornamented non-motile cells (30–32 mm diameter). Non-motile cells resembled the division cysts of *Scrippsiella hangoei* (J. Schiller) [47,48]. For each experiments testing the effect of any nitrogen source, the flasks were inoculated only with healthy motile cells. Our microscopic observations clearly showed that the cultures were dominated largely by motile cells (>95%) until the stationary phase, when non-motile cells appeared and settled onto the flask wall. Growth rate was calculated for the exponential phase only based on motile cells. PnTX-G and Chl *a* were measured at the exponential phase and at the beginning of the stationary phase, when motile cells still dominated.

### 3.2. Experimental Design

A culture of *V. rugosum* harvested in the exponential phase of growth was filtered on a GF/F filter to retain all the cells. Vegetative cells were then placed in the ENSW medium without the addition of nitrogen. Subsequently, cells were incubated during one week, after which cells were motile in the optimal physiological state and the nitrogen naturally present in the ENSW medium was completely depleted. At the beginning of the experiment, this culture was used to inoculate 250-mL sterile flasks containing various forms of nitrogen (nitrate, urea, and ammonium) to a final concentration of 110 μmol-N/L. This concentration was defined following a preliminary experiment where we determined the growth rate of *V. rugosum* in cultures with different nitrogen concentrations (55, 110, 220, and 549 μmol-N/L). Ammonium was the only nitrogen form toxic to *V. rugosum* at concentrations of 220 and 549 μmol-N/L. For each nitrogen source, the experiment was conducted in triplicate. During up to one month, we performed a cell count every 2–3 days to minimize interference with growth of the algae. Samples from cultures were collected during the exponential and stationary growth phases to analyze toxin and chlorophyll content and size.

### 3.3. Growth Rate Calculation

For all experiments, every day for two weeks, the experimental flasks were gently shaken and 500 μL representative samples were fixed using Lugol’s iodine solution. Cell concentration was monitored via direct microscopic counts using a Nageotte counting chamber. In accordance with Guillard [49], the specific growth rate μ (expressed in day^−1^) was calculated from the slope of a linear regression over the entire exponential phase of growth by the least square fit of a straight line to the data after logarithmic transformation; μ = (L_n_·N_t_ − L_n_·N_0_)/(t_1_ − t_0_) in units of day^−1^, where N_0_ and N_t_ represent the cell density in cells/mL at the start, t_0_, and end, t_1_, of the exponential phase, respectively.

### 3.4. Cell Diameter Measurement

The cell diameter was calculated as the average diameter of at least 30 cells using a FlowCam^®^ device (Fluid Imaging Technologies, Inc., Scarborough, ME, USA). The FlowCam^®^ is an instrument that combines the capabilities of a selective flow cytometer, microscopy (taking photos and biometrics), and fluorescence detectors. It analyzes particles or cells in a sample stream [50].

### 3.5. Nitrogen Content in the Cultures

The nitrogen concentration in the culture medium was measured every 5 days. Nitrate was measured using the method described by Collos [51] based on the absorption of nitrate in the UV. The sample was filtered on a membrane of 0.2 μm Pall Gelman Acrodisk (Pall Corporation, Port Washington, NY, USA) and no reagent was employed. The measurement was performed at 220 nm using a spectrophotometer Hitachi U-3000 (Hitachi High-Technologies, Velizy, France). The nitrate concentration was estimated by reference to a calibration curve prepared with a water solution of artificial sea water treated, as described above, with the following concentrations: 10, 20, 50, 100, and 200 μmol. Ammonium measurement was performed using the method described by Koroleff [52]. The determination of urea was performed using the method of Aminot and Kérouel [53].

### 3.6. Toxin Extraction and Quantification

For toxin analyses, we took a 20-mL sample in each culture at the exponential and stationary growth phases. The samples were centrifuged (3000 *g*, 15 min, 4 °C) and the supernatant removed carefully. Methanol (100%, 1 mL) was added to the remaining pellet and the sample stored at −20 °C until extraction of toxins [10]. Extraction of toxins was carried using two consecutive sonication steps for 10 min each, followed by filtration of extracts over a 0.2-μm membrane (Whatman Mini-UniPrep™, GE Healthcare Bio-Sciences, Pittsburgh, PA, USA) The filtered extracts were stored at −24 °C until quantification. Quantification of PnTX-G was carried out using liquid chromatography coupled to tandem mass spectrometry (LC-MS/MS) (SCIEX, Framingham, MA, USA), using external calibrants over the range from 0.5 to 100 ng/mL. A C8 column (Phenomenex, Torrance, CA, USA) was used at 25 °C for analysis (injection volume of 5 μL). The analysis was conducted at a flow rate of 0.8 mL/min [12].

### 3.7. Chlorophyll a Measurements

Chlorophyll *a* (Chl *a*) was determined by spectrofluorimetry LS50B (PerkinElmer, Waltham, MA, USA) using the method described by Neveux and Lantoine [54]. Cultures (15 or 20 mL) were filtered over GF/F filters (diameter 25 mm). Filters were stored at −24 °C until extraction of the pigments with aqueous acetone (90%). The extraction was carried out by allowing the filters to soak for 24 h at 4 °C after adding acetone (5 mL) and sonication for 10 s (twice). The samples were then centrifuged (2750 *g*, 4 °C for 15 min) and 3 mL were analyzed. Pigment concentration was expressed in pg/cell, using the cell concentration obtained by counting cells on the same day as sampling for chemical analyses.

## 4. Conclusions

*V. rugosum* could use osmotrophy to enhance nutrient supplies in a relatively nutrient poor habitat such as the Ingril lagoon (inorganic nitrogen concentration measured in 2012–2013 ranged between 0.02 and 6.75 μmol/L and 0.07 and 4.74 μmol/ L for nitrate and ammonium, respectively). The production of PnTx-G was significantly lower with urea as a nitrogen source, suggesting that nutritional conditions prevailing at the moment of the bloom could determine the cellular toxicity of *V. rugosum*. This could partially influence the toxin concentration measured in the contaminated mollusks. However, the accumulation, biotransformation, and depuration capacity of the bivalve also have to be considered.
